# Virburnum Prunifolium

**Published:** 1867-03

**Authors:** 


*Viburnum Prunifolium.*—(Black Haw.)

In the November number of this Journal Dr. D. L. Phares, of Newtonia, Mississippi, in an article headed "Lines on Indiginous Medicinal Plants," calls attention to the medicinal properties of the Black Haw.

We are again induced to call the attention of the profession to this comparatively new medicinal agent, that we may give our evidence as further proof of the favorable results claimed for it. Dr. Phares regards the Viburnum as a nervine, antispasmodic, astringent, diuretic, and tonic. He contends that in the nervous disorders incident to pregnancy and uterine troubles, such as cramps, palpitations, spasms, etc., it is a valuable remedy. He adds, "*that it is particularly valuable in preventing abortion and miscarriage, whether habitual or otherwise; whether threatened from accidental cause, or criminal drugging.*"

Within the past few weeks we have given the above remedy in two cases of threatened miscarriage, and in both with the most satisfactory results. The first case in which we administered it, was that of an exceedingly delicate lady, who, fourteen months previously, had miscarried between the fifth and sixth month. For a week or more preceding the administration of this agent, we had persevered in the usual remedies for threatened miscarriage, without accomplishing more than the mitigation of the pains.

For twenty-four hours before the use of the Viburnum, it required from one to two teaspoonsfull of the tincture of opium, every six or eight hours, to control the pains. In six hours after the first dose of the Viburnum, the pains were entirely arrested. As an evidence that the cessation was due to this agent, and not a mere coincidence, the infusion of Viburnum, through mistake, was suspended, with a return of the pains, which were again arrested by resuming the remedy. Anodynes, which were required in such large doses, before the administration of this new agent, have not been necessary since its use. The case, in every particular, is progressing favorably.

The second case, was a lady in her eighth month of pregnancy, who, for a week or ten days had been suffering occasional pains, with constant pain in the lumbar region, and cramps in the lower extremities, etc. Anodynes were administered, and sinapisms applied to the spine, with only temporary benefit; the pain and other troubles returning as soon as the effect of the remedies subsided. In this condition, the infusion of Viburnum was given, which promptly relieved all the trouble. She continues to take the remedy three times a day, and is progressing favorably. Another case similar to the last mentioned, with the addition of neuralgia of the face, was reported to me to-day, by a physician of the city, which was promptly relieved by the infusion of Viburnum.

The form in which we have used the Viburnum has been the infusion, or decoction of the bark. If the symptoms are urgent, we give from one to two ounces every two or three hours, until the pain is relieved, then lessen the dose, and lengthen the interval according to circumstances. As a preventive, we give an ounce of the infusion, three or four times a day.

